# ChIP-seq reveals broad roles of SARD1 and CBP60g in regulating plant immunity

**DOI:** 10.1038/ncomms10159

**Published:** 2015-12-18

**Authors:** Tongjun Sun, Yaxi Zhang, Yan Li, Qian Zhang, Yuli Ding, Yuelin Zhang

**Affiliations:** 1 Department of Botany, University of British Columbia, Vancouver, British Columbia, Canada V6T 1Z4; 2 National Institute of Biological Sciences, Beijing, 102206, China

## Abstract

Recognition of pathogens by host plants leads to rapid transcriptional reprogramming and activation of defence responses. The expression of many defence regulators is induced in this process, but the mechanisms of how they are controlled transcriptionally are largely unknown. Here we use chromatin immunoprecipitation sequencing to show that the transcription factors SARD1 and CBP60g bind to the promoter regions of a large number of genes encoding key regulators of plant immunity. Among them are positive regulators of systemic immunity and signalling components for effector-triggered immunity and PAMP-triggered immunity, which is consistent with the critical roles of SARD1 and CBP60g in these processes. In addition, SARD1 and CBP60g target a number of genes encoding negative regulators of plant immunity, suggesting that they are also involved in negative feedback regulation of defence responses. Based on these findings we propose that SARD1 and CBP60g function as master regulators of plant immune responses.

## Abstract (web summary)

SARD1 and CBP60g are two plant transcription factors that regulate salicylic acid biosynthesis in response to pathogens. Here, Sun *et al.* show that they bind a wide array of loci related to multiple defence signalling pathways suggesting a broader role as regulators of the plant immune response.

## Graphical abstract

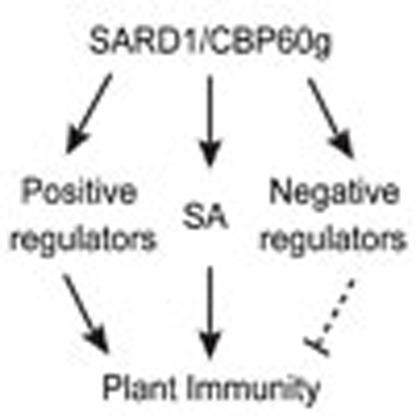

Plants use a multilayered defence system to combat microbial pathogens. At the front line, pattern recognition receptors on the plasma membrane recognize conserved features of microbes, collectively known as microbe-associated molecular patterns or pathogen-associated molecular patterns (PAMPs), to activate PAMP-triggered immunity (PTI)^1^. Most PAMP receptors belong to the receptor-like kinase and the receptor-like protein families. A second line of plant defence called effector-triggered immunity (ETI) relies on resistance (R) proteins that detect effector proteins secreted by pathogens to inhibit PTI (ref. 2). The majority of plant R proteins belong to the intracellular nucleotide-binding site (NB) leucine-rich repeats (LRR) protein family. Recognition of pathogens and activation of local defence responses further induce a secondary immune response in the distal part of plants termed systemic acquired resistance (SAR)^3^.

Salicylic acid (SA) is a signal molecule that plays key roles in local defence and SAR (ref. 4). *SALICYLIC ACID INDUCTION-DEFICIENT 2* (*SID2*) and *ENHANCED DISEASE SUSCEPTIBILITY 5* (*EDS5*) are required for pathogen-induced SA accumulation^5^,^6^. Mutations in *SID2* or *EDS5* block the accumulation of SA, resulting in enhanced susceptibility to pathogens and loss of SAR (refs 5, 6, 7). *SID2* encodes Isochorismate Synthase 1 (ICS1), which is a key enzyme in pathogen-induced SA synthesis^6^. *EDS5* encodes a transporter involved in exporting SA from chloroplast to cytoplasm^8^,^9^. Activation of defence gene expression and pathogen resistance by SA depends on the downstream component NON-EXPRESSOR OF PATHOGENESIS RELATED GENES1 (NPR1)^10^. Recent studies showed that NPR1 and its paralogs, NPR3 and NPR4, bind to SA and may function as SA receptors^11^,^12^.

Several genes encoding enzymes implicated in the synthesis of secondary metabolites have also been identified to be essential for SAR. Among them, *FLAVIN-DEPENDENT MONOOXYGENASE1* (*FMO1*) encodes a putative monooxygenase^13^,^14^,^15^, *AGD2-LIKE DEFENSE RESPONSE PROTEIN1* (*ALD1*) encodes an aminotransferase^16^, and *avrPphB SUSCEPTIBLE3* (*PBS3*) encodes a member of the firefly luciferase superfamily^17^,^18^,^19^. In *fmo1*, *ald1* and *pbs3* mutants, SAR is severely compromised^15^,^20^,^21^. ALD1 is involved in the synthesis of pipecolic acid, which contributes to the induction of SAR (ref. 22), while the chemicals synthesized by FMO1 and PBS3 remain to be determined.

Two pathogen-induced transcription factors, SAR DEFICIENT1 (SARD1) and CAM-BINDING PROTEIN 60-LIKE G (CBP60g), regulate the expression of *ICS1* and are required for pathogen induction of SA synthesis^23^,^24^,^25^. Following pathogen infection, SARD1 and CBP60g are recruited to the promoter of *ICS1* (ref. 24). In the *sard1 cbp60g* double mutant, induction of *ICS1* expression and SA synthesis is blocked^24^,^25^. SARD1 and CBP60g belong to the same protein family but are regulated differently, suggesting that they function in two parallel pathways to activate *ICS1* expression^23^,^24^. CBP60g, but not SARD1, can bind calmodulin. On the other hand, overexpression of *SARD1*, but not *CBP60g*, leads to constitutive activation of defence responses.

*Arabidopsis SNC2* encodes an receptor-like protein that is required for resistance against pathogenic bacteria *Pseudomonas syringae* pv *tomato* (*P.s.t.*) DC3000 and non-pathogenic bacteria *P. syringae* pv *tomato* DC3000 *hrcC* (refs 26, 27). A gain-of-function mutation in *snc2-1D* leads to constitutive activation of both SA-dependent and SA-independent defence pathways^26^. The *snc2-1D* mutant has small stature, accumulates high levels of salicylic acid, constitutively expresses *PATHOGENESIS-RELATED (PR)* genes, and exhibits enhanced pathogen resistance. From a suppressor screen of *snc2-1D npr1-1*, WRKY DNA-BINDING PROTEIN 70 (WRKY70) was identified as an essential regulator of the SA-independent pathway downstream of *snc2-1D* (ref. 26).

Here we report that SARD1 and CBP60g regulate not only the expression of *ICS1* and SA synthesis, but also the expression of *WRKY70* and the SA-independent defence pathway in *snc2-1D*. Chromatin immunoprecipitation (ChIP) analysis revealed that a large number of plant defence regulators including *WRKY70* are direct binding targets of SARD1 and CBP60g suggesting that SARD1 and CBP60g function as master regulators of plant defence responses.

## Results

### SARD1 and CBP60g are required for autoimmunity in *snc2-1D*

**Figure 1:**
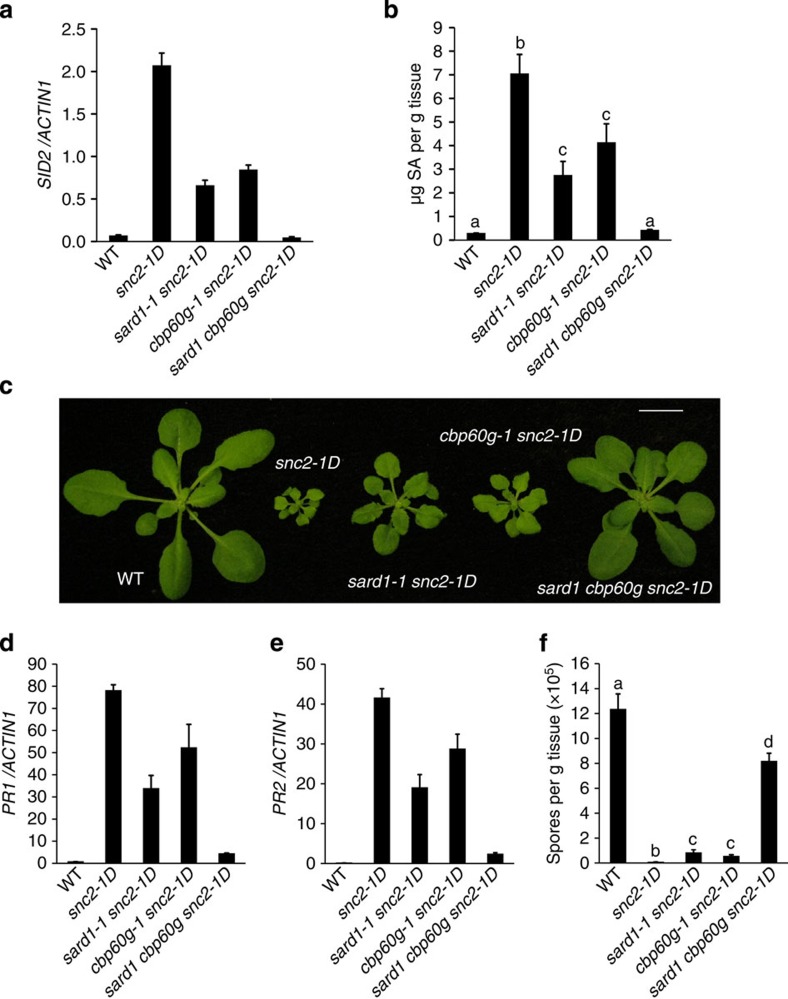
Mutations in *SARD1* and *CBP60g* largely suppress *snc2-1D*-mediated autoimmunity. (**a**) *SID2* expression in wild-type Col-0, *snc2-1D*, *sard1-1 snc2-1D*, *cbp60g-1 snc2-1D* and *sard1-1 cpb60g-1 snc2-1D* mutant plants. The expression was normalized with *ACTIN1*. Bars represent means±s.d (*n*=3). (**b**) Free SA levels in the indicated genotypes. Bars represent means±s.d. (*n*=4). Statistical differences among the samples are labelled with different letters (ANOVA, *P*<0.01). (**c**) Morphology of 3-week-old soil-grown plants of the indicated genotypes. Bar represents 1 cm. (**d**,**e**) Expression levels of *PR1* (**d**) and *PR2* (**e**) in the indicated genotypes as normalized by *ACTIN1*. Bars represent means±s.d. (*n*=3). (**f**) Quantification of *H. arabidopsidis* Noco2 sporulation on the indicated genotypes. Bars represent means±s.d. (*n*=4). Statistical differences among the samples are labelled with different letters (*P*<0.01, one-way ANOVA; *n*=3). Plants were grown on soil at 23 °C and assayed 3 weeks after planting. ANOVA, analysis of variance.

To determine whether the increased SA synthesis in *snc2-1D* mutant plants is dependent on SARD1 and CBP60g, we crossed *sard1-1* and *cbp60g-1* into *snc2-1D* to obtain the *sard1-1 snc2-1D* and *cbp60g-1 snc2-1D* double mutants and the *sard1-1 cbp60g-1 snc2-1D* triple mutant. Quantitative reverse transcription PCR (RT–PCR) analysis showed that the expression of *ICS1* in *snc2-1D* is much higher than in wild type, but the increased expression of *ICS1* is blocked in the *sard1-1 cbp60g-1 snc2-1D* triple mutant (Fig. 1a). Consistent with the expression levels of *ICS1*, increased accumulation of SA in *snc2-1D* is also suppressed in the triple mutant (Fig. 1b).

The *sard1-1 snc2-1D* and *cbp60g-1 snc2-1D* double mutants have similar morphology as *snc2-1D* and are only slightly bigger than *snc2-1D* (Fig. 1c). Surprisingly, the mutant morphology of *snc2-1D* is almost completely suppressed in the *sard1-1 cbp60g-1 snc2-1D* triple mutant (Fig. 1d). Quantitative RT–PCR analysis showed that the expression levels of defence marker genes *PR1* and *PR2* are slightly lower in the double mutants but are markedly reduced in the triple mutant compared with *snc2-1D* (Fig. 1d,e). In addition, the enhanced resistance to *Hyaloperonospora arabidopsidis* Noco2 in *snc2-1D* is partially reduced in the double mutants and almost completely lost in the triple mutant (Fig. 1f). As blocking SA accumulation by *eds5-3* has very little effect on the morphology, *PR2* expression and resistance to *H. arabidopsidis* Noco2 in *snc2-1D* (ref. 26), these data suggest that SARD1 and CBP60g also regulate SA-independent pathways in *snc2-1D*.

### SARD1 and CBP60g regulate the expression of *WRKY70*

In *sard1 cpb60g* mutant plants expressing the SARD1-HA fusion protein under its native promoter, pathogen-induced *ICS1* expression was restored to similar level as in the *cbp60g* single mutant, suggesting that SARD1-HA functions similarly as wild-type SARD1 protein (Supplementary Fig. 1). To identify genes targeted by SARD1, ChIP was carried out on transgenic plants expressing a SARD1-HA fusion protein under its own promoter using an anti-HA antibody. The immunoprecipitated DNA was sequenced by Illumina sequencing. Analysis of the ChIP-sequencing (ChIP-seq) data showed a × 20 genome coverage.

Sequence coverage at each position on the genome was plotted to identify peaks in the *Arabidopsis* genome. Analysis of peaks in the genic region showed that most sequence peaks are located in the 1.5 kb region upstream of the translation start site, which includes the 5'-UTRs and promoter regions. After removing genes that showed similar sequence peaks in the negative control, peaks with heights of 90 or greater were found in the introns of 84 genes, the 3'-UTRs of 60 genes and the 1.5 kb region upstream of the translation start sites of 1,902 genes. We focused our analysis on the group containing peaks with heights of 90 or greater in the 1.5 kb region upstream of the translation start sites (Supplementary Data 1), because it contains many genes encoding known regulators of plant defence that are strongly induced by pathogen infection (Table 1). Distribution of sequence reads in the promoter and coding regions of these known defence regulators are shown in Supplementary Fig. 2.

**Figure 2:**
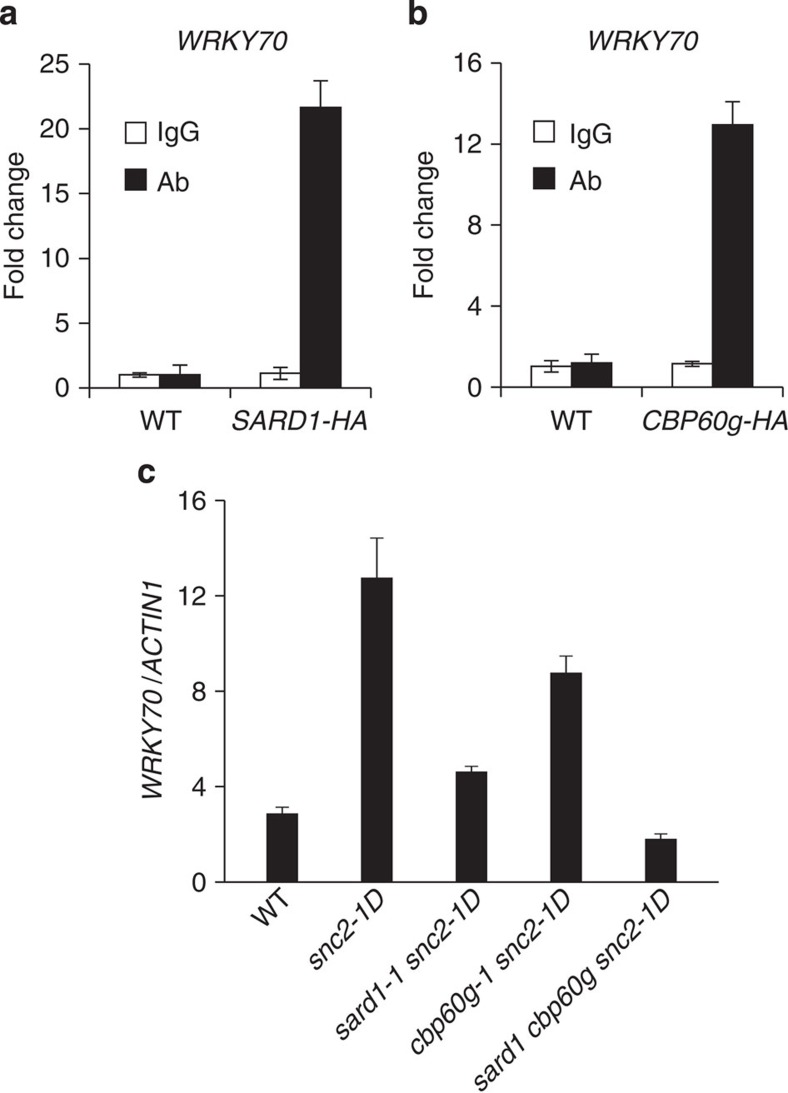
SARD1 and CBP60g regulate the expression of *WRKY70*. (**a**,**b**) Binding of SARD1 (**a**) and CBP60g (**b**) to the promoter of *WRKY70* as determined by ChIP-PCR. Leaves of 25-day-old wild-type plants and *SARD1-HA* (**a**) or *CBP60g-HA* (**b**) transgenic plants were infiltrated with *P.s.m.* ES4326 (OD_600_=0.001) 24 h before collecting and cross-linking with 1% formaldehyde. SARD1-HA and CBP60g-HA chromatin complexes were immunoprecipitated with an anti-HA antibody and protein A agarose beads. Negative control reactions were performed in parallel using immunoglobin G (IgG). Immunoprecipitated DNA samples were quantified by real-time qPCR using primers specific to *WRKY70* promoter. ChIP results are presented as fold changes by dividing signals from ChIP with the anti-HA antibody by IgG controls, which are set as one. Bars represent means±s.d. (*n*=3). (**c**) *WRKY70* expression in the indicated genotypes as normalized with *ACTIN1*. Bars represent means±s.d. (*n*=3). qPCR, quantitative PCR.

One of the candidate target genes of SARD1 identified by ChIP-seq is *WRKY70* (Table 1), which is known to regulate SA-independent defence responses in *snc2-1D* (ref. 26). Quantitative PCR analysis of the DNA immunoprecipitated by the anti-HA antibody confirmed that *WRKY70* is a binding target of SARD1 (Fig. 2a). In *sard1 cpb60g* mutant plants expressing the CBP60g-HA fusion protein under its native promoter, pathogen-induced *ICS1* expression was restored to similar level as in the *sard1* single mutant, suggesting that CBP60g-HA functions similarly as wild-type protein (Supplementary Fig. 1). To determine whether *WRKY70* is also a binding target of CBP60g, we carried out ChIP-PCR experiments on transgenic plants expressing a CBP60g-HA fusion protein under its own promoter using the anti-HA antibody. As shown in Fig. 2b, CBP60g is also targeted to the promoter region of *WRKY70*.

Next we analysed the expression of *WRKY70* in *snc2-1D*, *sard1-1 snc2-1D*, *cbp60g-1 snc2-1D* and *sard1-1 cbp60g-1 snc2-1D* mutant plants. As shown in Fig. 2c, WRKY70 is expressed at a considerably higher level in *snc2-1D* than in wild type. The expression of *WRKY70* is slightly lower in *cbp60g-1 snc2-1D* and clearly reduced in *sard1-1 snc2-1D* compared with *snc2-D*. However, it is further reduced to below wild-type level in the *sard1-1 cbp60g-1 snc2-1D* triple mutant (Fig. 2c). These data suggest that SARD1 and CBP60g have overlapping functions in regulating the expression of *WRKY70* and that reduced expression of WRKY70 is at least partly responsible for the suppression of the *snc2-1D*-mediated SA-independent constitutive defence responses in the *sard1-1 cbp60g-1 snc2-1D* triple mutant.

### SARD1 and CBP60g regulate the expression of *EDS5* and *NPR1*

**Figure 3:**
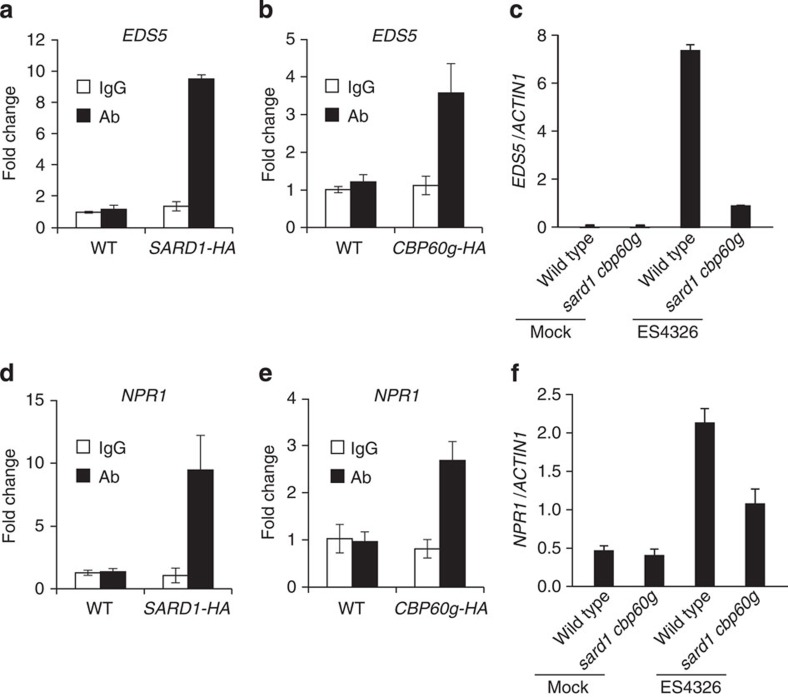
*EDS5* and *NPR1* are direct binding targets of SARD1 and CBP60g. (**a**,**b**) Recruitment of SARD1-HA (**a**) and CBP60g-HA (**b**) to *EDS5* promoter after *P.s.m.* ES4326 infection as determined by ChIP-PCR. ChIP was performed as described in Fig. 2. Real-time PCR was carried out using primers specific to *EDS5* promoter. ChIP results are presented as fold changes by dividing signals from ChIP with the anti-HA antibody by those of IgG controls, which are set as one. Bars represent means±s.d. (*n*=3). (**c**) Induction of *EDS5* expression in wild type and *sard1-1 cpb60g-1* by *P.s.m.* ES4326. Leaves of 25-day-old plants were infiltrated with *P.s.m.* ES4326 (OD_600_=0.001) or 10 mM MgCl_2_ (mock) 12 h before collection for RT–PCR analysis. Bars represent means±s.d. (*n*=3). (**d**,**e**) Recruitment of SARD1-HA (**d**) and CBP60g-HA (**e**) to the promoter of *NPR1* after treatment with *P.s.m.* ES4326. ChIP and data analysis were carried out similarly as in **a** and **b**. Bars represent means±s.d. (*n*=3). (**f**) Induction of *NPR1* expression by *P.s.m.* ES4326 in wild-type and *sard1-1 cpb60g-1* double mutant plants. Samples were collected 12 h after infiltration with *P.s.m.* ES4326 (OD_600_=0.001) or 10 mM MgCl_2_ (mock). Bars represent means±s.d. (*n*=3).

*EDS5* is involved in pathogen-induced SA synthesis^5^,^7^. Analysis of the SARD1 ChIP-seq data revealed that *EDS5* is a potential target gene of SARD1 as well (Table 1). A peak with a height of 110 was identified ∼700 bp upstream of the translation start site of *EDS5*. ChIP-PCR experiments confirmed that SARD1 is targeted to the promoter region of *EDS5* (Fig. 3a). Further ChIP-PCR analysis showed that CBP60g also binds to the promoter region of *EDS5* (Fig. 3b). To determine whether SARD1 and CBP60g are required for the induction of *EDS5* by *Pseudomonas syringae* pv. *maculicola* (*P.s.m.*) ES4326, we compared the expression levels of *EDS5* in wild type and *sard1-1 cbp60g-1* plants. As shown in Fig. 3c, induction of *EDS5* by *P.s.m.* ES4326 is greatly reduced in the *sard1-1 cbp60g-1* double mutant. These data suggest that SARD1 and CBP60g directly regulate pathogen-induced expression of *EDS5*.

Another candidate target gene of SARD1 identified by ChIP-seq is *NPR1*, which encodes a putative SA receptor^11^. A peak with a height of 163 was identified ∼100 bp upstream of the translation start site of *NPR1* (Table 1). Binding of SARD1 to the promoter region of *NPR1* was confirmed by ChIP-PCR (Fig. 3d). As shown in Fig. 3e, CBP60g is also targeted to the promoter region of *NPR1*. Analysis of the expression levels of *NPR1* in wild type and *sard1-1 cbp60g-1* plants showed that induction of *NPR1* by *P.s.m.* ES4326 is compromised in the *sard1-1 cbp60g-1* double mutant (Fig. 3f). These data suggest that SARD1 and CBP60g also regulate pathogen-induced expression of *NPR1*.

### Multiple SAR regulators are targets of SARD1 and CBP60g

**Figure 4:**
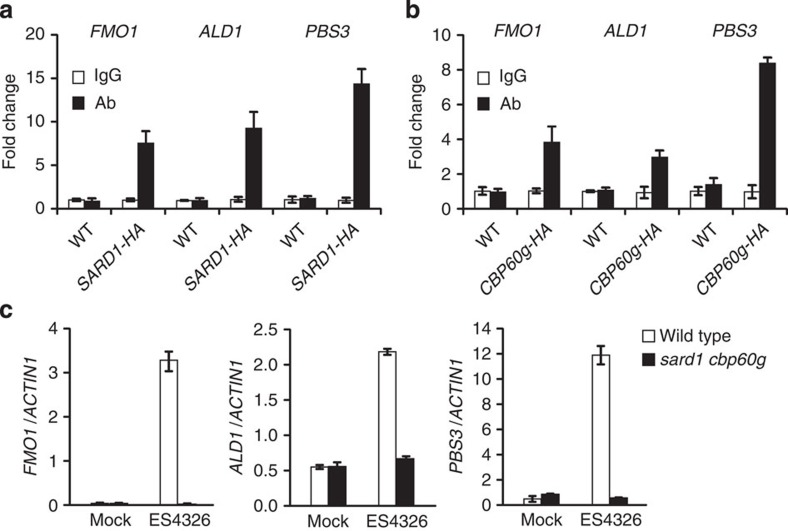
SARD1 and CBP60g target genes essential for SAR. (**a**,**b**) Binding of SARD1-HA (**a**) and CBP60g-HA (**b**) to the promoter regions of *FMO1*, *ALD1* and *PBS3* following infection by *P.s.m.* ES4326 as determined by ChIP-PCR. ChIP was performed as described in Fig. 2. Real-time PCR was carried out using gene-specific primers. ChIP results are presented as fold changes by dividing signals from ChIP with the anti-HA antibody by those of the IgG control, which are set as one. Bars represent means±s.d. (*n*=3). (**c**) Induction of the expression of *FMO1*, *ALD1* and *PBS3* by *P.s.m.* ES4326 infection. Samples were collected 12 h after inoculation with *P.s.m.* ES4326 (OD_600_=0.001) or 10 mM MgCl_2_ (mock). Expression levels were normalized with *ACTIN1*. Bars represent means±s.d. (*n*=3).

In addition to *EDS5* and *NPR1*, three other genes required for SAR, *FMO1*, *ALD1* and *PBS3*, were identified as candidate target genes of SARD1 from the ChIP-seq data. The height of the peaks identified in the promoter regions of *FMO1*, *ALD1* and *PBS3* are 99, 138 and 199, respectively (Table 1). Binding of SARD1 to the promoters of these three genes was confirmed by ChIP-PCR experiments (Fig. 4a). Further ChIP-PCR analysis showed that CBP60g also binds to the promoters of these genes (Fig. 4b). Consistent with data from previous gene expression studies^25^,^28^, we also observed dramatic reduction in bacteria-induced expression of *FMO1*, *ALD1* and *PBS3* in the *sard1-1 cbp60g-1* double mutant (Fig. 4c). These data suggest that SARD1 and CBP60g directly regulate the expression of *FMO1*, *ALD1* and *PBS3* in plant defence responses.

### SARD1 and CBP60g target positive regulators of ETI

**Figure 5:**
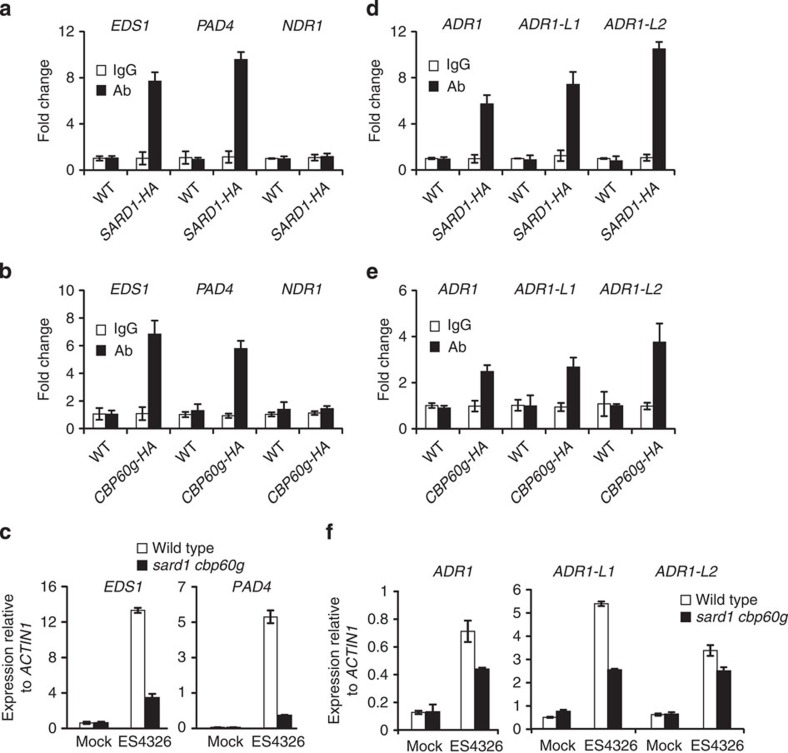
SARD1 and CBP60g regulate the expression of positive regulators of ETI. (**a**,**b**) Binding of SARD1-HA (**a**) and CBP60g-HA (**b**) to promoter regions of *EDS1* and *PAD4* following infection by *P.s.m.* ES4326 as determined by ChIP-qPCR. *NDR1* is not a direct target of SARD1 or CPB60g and is used as negative control. ChIP was performed as described in Fig. 2. Real-time PCR was carried out using primers specific to *EDS1*, *PAD4* and *NDR1* promoters. ChIP results are presented as fold changes by dividing signals from ChIP with the anti-HA antibody by those of the IgG controls, which are set as one. Bars represent means±s.d. (*n*=3). (**c**) Induction of *EDS1* and *PAD4* expression in wild type and *sard1-1 cpb60g-1* plants after *P.s.m.* ES4326 infection. Expression levels were normalized with *ACTIN1*. Bars represent means±s.d. (*n*=3). (**d**,**e**) Binding of SARD1-HA (**d**) and CBP60g-HA (**e**) to the promoter regions of *ADR1*, *ADR1-L1* and *ADR1-L2* following infection by *P.s.m.* ES4326 as determined by ChIP-PCR. ChIP and data analysis were carried out similarly as in **a** and **b**. Bars means±s.d. (*n*=3). (**f**) Induction of *ADR1*, *ADR1-L1* and *ADR1-L2* expression by *P.s.m.* ES4326 as determined by real-time RT–PCR. Samples were collected 12 h after inoculation with *P.s.m.* ES4326 (OD_600_=0.001) or 10 mM MgCl_2_ (mock). Expression levels were normalized with *ACTIN1*. Bars represent means±s.d. (*n*=3). qPCR, quantitative PCR.

*ENHANCED DISEASE SUSCEPTIBILITY 1* (*EDS1*) and *PHYTOALEXIN DEFICIENT* 4 (*PAD4*) encode positive regulators of defence responses activated by TIR-NB-LRR R proteins^29^,^30^,^31^,^32^,^33^. *NDR1* is required for defence responses activated by CC-NB-LRR R proteins^29^,^34^. *EDS1* and *PAD4*, but not *NDR1*, were identified as candidate target genes of SARD1 by ChIP-seq (Table 1). The height of the peaks identified in the promoter regions of *EDS1* and *PAD4* are 258 and 137, respectively. ChIP-PCR experiments showed that SARD1 was targeted to the promoter regions of *EDS1* and *PAD4*, but not *NDR1* (Fig. 5a). In addition, CBP60g is also targeted to the promoters of *EDS1* and *PAD4*, but not *NDR1* (Fig. 5b). Quantitative RT–PCR was subsequently carried out to determine whether induction of the expression of *EDS1* and *PAD4* by bacterial infections is dependent on SARD1 and CBP60g. As shown in Fig. 5c, induction of *EDS1* and *PAD4* by *P.s.m.* ES4326 is markedly reduced in the *sard1-1 cbp60g-1* double mutant. These data suggest that induction of *EDS1* and *PAD4* following pathogen infection is directly regulated by SARD1 and CBP60g.

*ADR1*, *ADR-L1* and *ADR-L2* encode three closely related CC-NB-LRR proteins required for immunity mediated by TIR-NB-LRR R proteins RPP2 and RPP4 (ref. 35). They were also identified as candidate target genes of SARD1 by ChIP-seq (Table 1). The heights of the peaks identified in the promoter regions of *ADR1*, *ADR-L1* and *ADR-L2* are 117, 324 and 230, respectively. ChIP-PCR analysis confirmed that SARD1 binds to the promoter regions of these three genes (Fig. 5d). ChIP-PCR experiments showed that CBP60g is also targeted to the promoter regions of *ADR1*, *ADR-L1* and *ADR-L2* (Fig. 5e). As shown in Fig. 5f, the expression of *ADR1*, *ADR-L1* and *ADR-L2* is induced by *P.s.m.* ES4326 and the induction is partially dependent on SARD1 and CBP60g. These data suggest that SARD1 and CBP60g are likely to directly regulate the expression of *ADR1*, *ADR-L1* and *ADR-L2* in plant defence.

### SARD1 and CBP60g play critical roles in PTI

**Figure 6:**
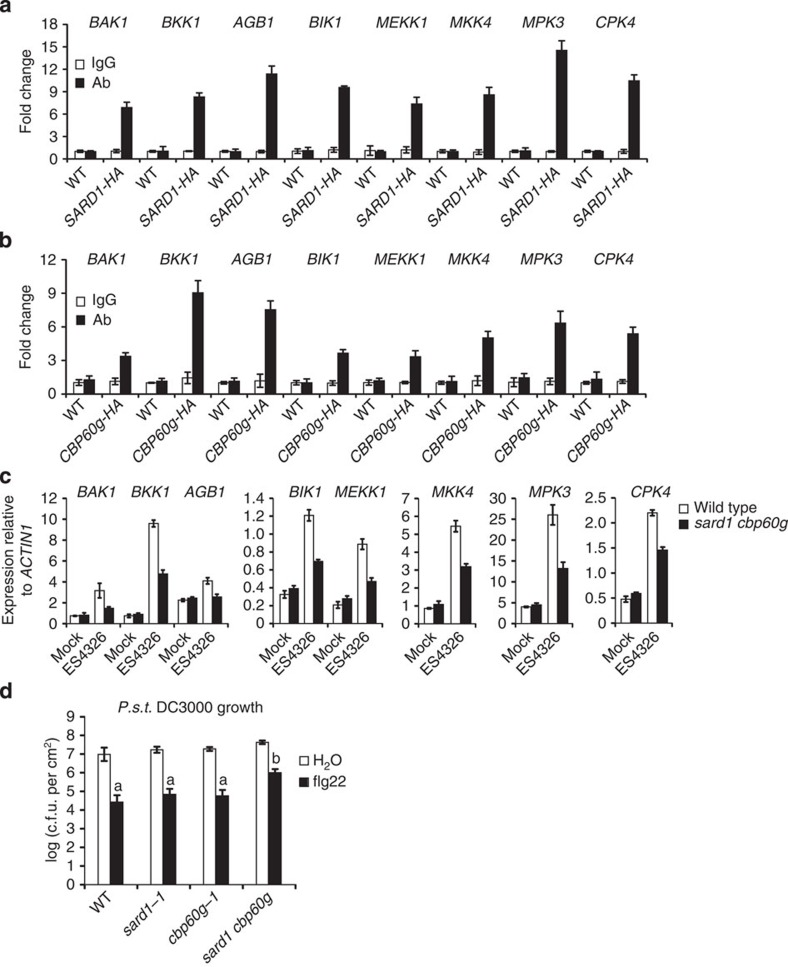
SARD1 and CBP60g modulate the expression of signalling components of PTI and contribute to flg22-induced resistance to *P.s.t.* DC3000. (**a**,**b**) Binding of SARD1-HA (**a**) and CPB60g-HA (**b**) to the promoter regions of *BAK1*, *BKK1*, *AGB1*, *BIK1*, *MEKK1*, *MKK4*, *MPK3* and *CPK4* following *P.s.m.* ES4326 infection as determined by ChIP-PCR. ChIP was performed as described in Fig. 2. Real-time PCR was carried out using gene-specific primers. ChIP results are presented as fold changes by dividing signals from ChIP with the anti-HA antibody by those of the IgG controls, which are set as one. Bars represent means±s.d. (*n*=3). (**c**) Induction of *BAK1*, *BKK1*, *AGB1*, *BIK1*, *MEKK1*, *MKK4*, *MPK3* and *CPK4* expression by *P.s.m.* ES4326 as determined by real-time RT–PCR. Samples were collected 12 h after inoculation with *P.s.m.* ES4326 (OD_600_=0.001) or 10 mM MgCl_2_ (mock). Expression levels of the genes were normalized with *ACTIN1*. Bars represent means±s.d. (*n*=3). (**d**) flg22-induced resistance to *P.s.t* DC3000 on the indicated genotypes. Four-week-old plants were infiltrated with 1 μM flg22 or H_2_O 1 day before inoculation with *P.s.t.* DC3000 (OD_600_=0.001). Bacterial growth was determined 3 days post inoculation. Bars represent means±s.d. (*n*=5). Statistical differences among the samples are labelled with different letters (ANOVA, *P*<0.001). ANOVA, analysis of variance; c.f.u., colony-forming unit; *P.s.t*., *P. syringae* pv *tomato*.

Among the candidate target genes of SARD1 identified by ChIP-seq, eight genes including *BAK1*, *BKK1*, *AGB1*, *BIK1*, *MEKK1*, *MKK4*, *MPK3* and *CPK4* (Table 1) were previously shown to encode positive regulators of PTI (refs 36, 37, 38, 39, 40, 41, 42, 43, 44, 45, 46, 47, 48). Binding of SARD1 to the promoter regions of these genes was further confirmed by ChIP-PCR (Fig. 6a). In addition, CBP60g is also targeted to the promoter regions of these genes (Fig. 6b). As shown in Fig. 6c, expression of *BAK1*, *BKK1*, *AGB1*, *BIK1*, *MEKK1*, *MKK4*, *MPK3* and *CPK4* is induced *P.s.m.* ES4326 and the induction is reduced in the *sard1-1 cbp60g-1* double mutant, suggesting that SARD1 and CBP60g may directly regulate their expression in plant defence responses.

To test whether SARD1 and CBP60g are required for PTI, we analysed bacterial growth in wild type, *sard1-1*, *cbp60g-1* and *sard1-1 cbp60g-1* plants pretreated with flg22, a peptide from bacterial flagellin that is recognized by *FLAGELLIN-SENSITIVE2* (FLS2) (ref. 49). As shown in Fig. 6d, flg22-induced resistance to *P. syringae* pv *tomato* DC3000 is not obviously affected in the *sard1-1* and *cbp60g-1* single mutants, but clearly reduced in the *sard1-1 cbp60g-1* double mutant, suggesting that SARD1 and CBP60g contribute to PTI.

### SARD1 and CBP60g target negative regulators of defence

**Figure 7:**
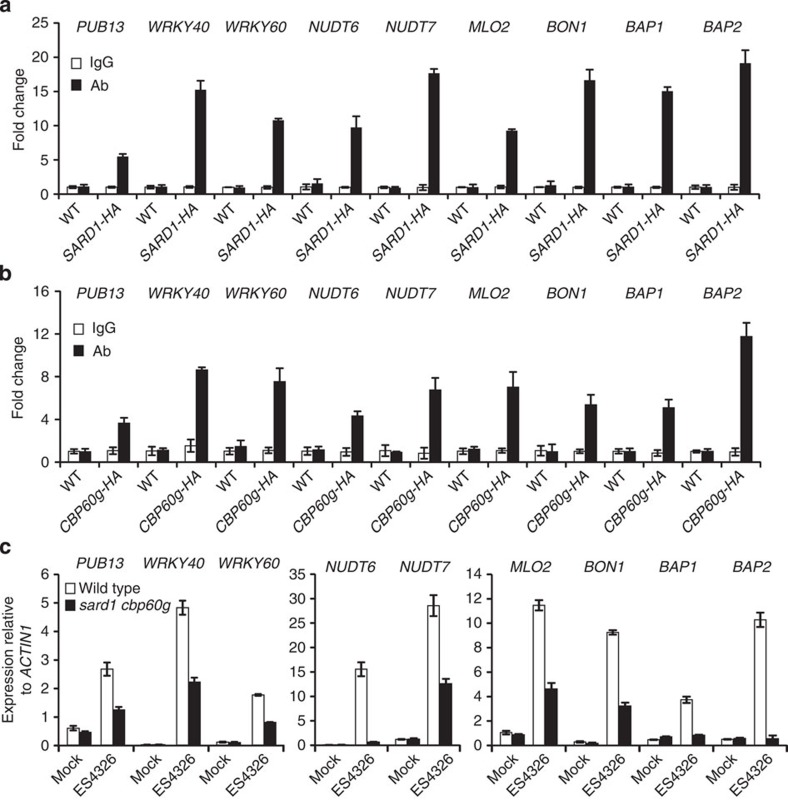
SARD1 and CBP60g modulate the expression of genes encoding negative regulators of plant defence. (**a**,**b**) Recruitment of SARD1-HA (**a**) and CBP60g-HA (**b**) to the promoter regions of *PUB13*, *WRKY40*, *WRKY60*, *NUDT6*, *NUDT7*, *MLO2*, *BON1*, *BAP1* and *BAP2* following *P.s.m.* ES4326 infection as determined by ChIP-PCR. ChIP was performed as described in Fig. 2. Real-time PCR was carried out using gene-specific primers. ChIP results are presented as fold changes by dividing signals from ChIP with the anti-HA antibody by those of the IgG controls, which are set as one. Bars represent means±s.d. (*n*=3). (**c**) Induction of *PUB13*, *WRKY40*, *WRKY60*, *NUDT6*, *NUDT7*, *MLO2*, *BON1*, *BAP1* and *BAP2* genes in wild type and *sard1-1 cpb60g-1* by *P.s.m.* ES4326. Samples were collected 12 h after inoculation with *P.s.m.* ES4326 (OD_600_=0.001) or 10 mM MgCl_2_ (mock). Expression levels of the genes were normalized with *ACTIN1*. Bars represent means±s.d. (*n*=3).

Analysis of the SARD1 ChIP-seq data also identified a number of negative regulators of plant immunity including *PUB13*, *WRKY40*, *WRKY60*, *NUDT6*, *NUDT7*, *MLO2*, *BON1*, *BAP1* and *BAP2* as candidate target genes of SARD1 (Table 1). Binding of SARD1 to the promoter regions of *PUB13*, *WRKY40*, *WRKY60*, *NUDT6*, *NUDT7*, *MLO2*, *BON1*, *BAP1* and *BAP2* was confirmed by ChIP-PCR analysis (Fig. 7a). In addition, CBP60g was also found to target the promoter regions of these nine genes (Fig. 7b). Quantitative RT–PCR analysis showed that the expression of *PUB13*, *WRKY40*, *WRKY60*, *NUDT6*, *NUDT7*, *MLO2*, *BON1*, *BAP1* and *BAP2* are all induced by *P.s.m.* ES4326 and the induction is either reduced or blocked in the *sard1-1 cbp60g-1* double mutant (Fig. 7c). These data suggest that SARD1 and CBP60g regulate the expression of these negative regulators of plant immunity during plant defence.

### SARD1 regulates gene expression through the GAAATTT element

Previously, we showed that SARD1 and CBP60g bind preferentially to the oligonucleotide probe GAAATTTTGG (ref. 24). Bioinformatics analysis showed that the GAAATTT motif within this probe is over-represented in the promoters of the genes with SARD1 and CBP60g-dependent expression^25^. Analysis of the 1,902 candidate target genes of SARD1 in Supplementary Data 1 showed that the GAAATTT motif is also over-represented in the promoter regions of this group of genes (*P*<10^−15^, Fisher's exact test). This motif is over-represented in the promoter regions of 29 confirmed target genes of SARD1 and CBP60g listed in Table 1 (*P*<0.005, Fisher's exact test) as well. However, not every gene in this group contains this motif in their promoter region. It is likely SARD1 and CBP60g can also bind to certain variants of the GAAATTT motif. Interestingly, a closely related sequence motif, G(A/T)AATT(T/G), was identified as a conserved motif (*P*<10^−25^, Fisher's exact test) among the sequence peaks of genes in Supplementary Data 1 using the motif discovery algorithm DREME.

**Figure 8:**
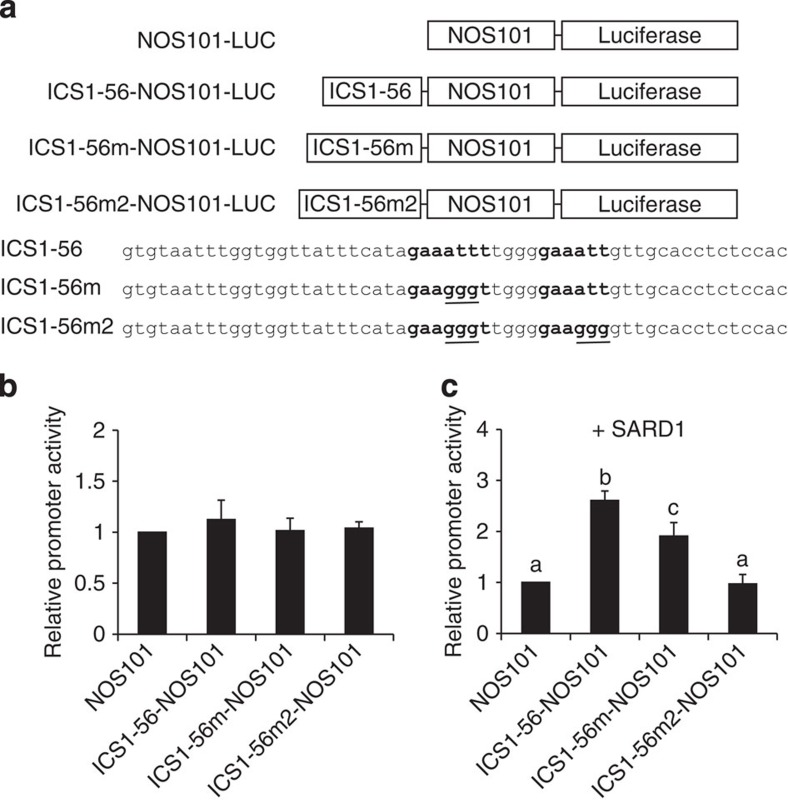
SARD1 activates reporter gene expression through the GAAATTT motif. (**a**) Reporter constructs used in the promoter activity assay. NOS101, a basal promoter of the nopaline synthase gene (−101 to +4); ICS1-56, a 56 bp fragment from the ChIP-Seq peak region in *ICS1* promoter; ICS1-56m and ICS1-56m2, mutant versions of ICS1-56 with mutations (underlined) in the GAAATTT or GAAATT motif. The GAAATTT and GAAATT sequences are bolded. (**b**) Luciferase activities in protoplasts transformed with individual reporter constructs. (**c**) Luciferase activities in protoplasts transformed with individual reporter constructs together with a *35S-SARD1* plasmid. A *35S-Renilla Luciferase* construct was included in all assays as an internal transformation efficiency control. The activities of luciferase were normalized with the expression of the Renilla luciferase and compared with the value obtained from protoplasts transfected with *NOS101-LUC* construct, which was set as 1. The error bars represent means±s.d. from three biological replicates.

To test whether SARD1 activates its target gene expression through the GAAATTT motif, we made a construct expressing the luciferase reporter gene under the control of a 56 bp fragment from the ChIP-Seq peak region in the promoter of *ICS1*, which contains a GAAATTT and a related GAAATT motif (Fig. 8a). Two additional constructs containing mutations in these two motifs were also created to determine whether they are required for activation of reporter gene expression by the 56 bp fragment. These reporter gene constructs were transformed into *Arabidopsis* protoplasts to exam the luciferase reporter expression levels. As shown in Fig. 8b, all three constructs expressed similar levels of luciferase as the original NOS101-Luc vector, suggesting that the 56 bp promoter fragment cannot activate luciferase expression on its own in protoplast transient assays. However, when the luciferase reporter gene constructs were co-transformed together with a plasmid expressing the SARD1 protein into protoplasts, luciferase expression was much higher in samples transformed with the construct containing the wild-type 56 bp promoter fragment compared with samples transformed with the NOS101-Luc vector (Fig. 8c). In comparison, samples transformed with the construct carrying mutations in the GAAATTT motif exhibited significantly reduced luciferase activity. When both the GAAATTT and GAAATT motifs were mutated, the luciferase activity was further reduced to a level similar to that in the NOS101-Luc vector control. Together, these data suggest that SARD1 activates gene expression through GAAATTT or similar DNA sequence elements.

## Discussion

SA functions as a key signalling molecule in SAR. SARD1 and CBP60g have previously been shown to regulate pathogen-induced SA synthesis^23^,^24^,^25^. In this study, we showed that, in addition to *ICS1*, the expression of another regulator of SA synthesis, *EDS5*, is also likely controlled by SARD1 and CBP60g. *NPR1*, a gene required for the perception of SA by plants, is a target of SARD1 and CBP60g as well. Moreover, SARD1 and CBP60g also regulate pathogen-induced expression of several other genes required for SAR. Both SARD1 and CBP60g are targeted to the promoter regions of *FMO1*, *ALD1* and *PBS3* and induction of these genes by *P.s.m.* ES4326 is markedly reduced in the *sard1 cbp60g* double mutant. These data suggest that SARD1 and CBP60g function in coordinating the induction of SAR regulators during plant defence.

Several defence regulators that function upstream of SA synthesis are also regulated by SARD1 and CBP60g. Both PAD4 and EDS1 are required for pathogen-induced SA synthesis^32^,^50^. SARD1 and CBP60g are targeted to their promoters and are required for their induction by *P.s.m.* ES4326. In addition, *ADR1*, *ADR1-L1* and *ADR1-L2*, three helper R genes required for pathogen-induced SA synthesis^35^, are also targets of SARD1 and CBP60g. Regulation of the induction of *PAD4*, *EDS1*, *ADR1*, *ADR1-L1* and *ADR1-L2* by SARD1 and CBP60g may play critical roles in promoting SA synthesis during pathogen infection.

We also found that SARD1 and CBP60g function downstream of the receptor-like protein SNC2 to regulate both SA-dependent and SA-independent defence pathways. SARD1 and CBP60g are required for the increased expression of *ICS1* and SA synthesis in *snc2-1D*. Regulation of the SA-independent defence pathway by SARD1 and CBP60g appears to be at least partly through their control of *WRKY70* expression, a key regulator of the SA-independent defence responses in *snc2-1D* (ref. 26), as both SARD1 and CBP60g are targeted to the promoter of *WRKY70* and are required for the induction of *WRKY70* in *snc2-1D*.

Furthermore, a large number of genes encoding regulatory components of PTI are binding targets of SARD1 and CBP60g and require SARD1 and CBP60g for induction by bacterial infection. Among them, BAK1 and BKK1 serve as co-receptors of FLS2 and EF-TU RECEPTOR (EFR) (refs 36, 37, 38). BIK1 and AGB1 function downstream of multiple PAMP receptors to regulate ROS production and defence against pathogen infection^39^,^40^,^41^,^42^,^43^,^44^,^45^. MEKK1, MKK4 and MPK3 are components of MAP kinase cascades downstream of PAMP receptors^47^,^51^,^52^,^53^,^54^. CPK4 was identified as a calcium dependent protein kinase downstream of FLS2 (ref. 48). The critical role of SARD1 and CBP60g in PTI was further confirmed by the attenuation of flg22-induced pathogen resistance in the *sard1 cbp60g* double mutant.

In addition to upregulation of positive regulators, negative regulators are often induced during plant defence as well. Induction of negative regulators is critical for feedback inhibition of defence responses to prevent uncontrolled activation, which may lead to autoimmunity. We showed that a number of negative regulators of plant immunity including *PUB13*, *WRKY40*, *WRKY60*, *NUDT6*, *NUDT7*, *MLO2*, *BON1*, *BAP1* and *BAP2* are also targets of SARD1 and CBP60g. Among them, PUB13 is a U-box/ARM E3 ubiquitin ligase that regulates cell death as well as degradation of FLS2 after flagellin induction^55^,^56^; WRKY40 and WRKY60 function redundantly with their close homologue, WRKY18, to repress basal defence^57^,^58^; NUDT6 and NUDT7 are two Nudix domain-containing proteins that negatively regulate EDS1-dependent immune responses^13^,^59^,^60^; MLO2 functions as a negative regulator of resistance to powdery mildew^61^; BON1 functions as a negative regulator of immunity mediated by the TIR-NB-LRR R protein SNC1 (ref. 62); BAP1 and BAP2 encode two C2 domain-containing proteins that negatively regulate programmed cell death^63^,^64^. All these genes are induced following infection by *P.s.m.* ES4326 and their induction requires SARD1 and CBP60g, suggesting that SARD1 and CBP60g also play an important role in the negative feedback regulation of plant defence.

Bioinformatics analysis has previously been used to analyze genes that are co-expressed with a group of SARD1/CBP60g-dependent genes^28^. Four genes including *AGP5*, *At5g52760*, *CML46* and *CML47* that form a small cluster with *SARD1* and *ICS1* were identified as candidate target genes of SARD1 and CBP60g. These genes are also identified as binding targets of SARD1 in our ChIP-seq data (Supplementary Data 1). *EDS1* and *PAD4* were also found to cluster with *ICS1* in the co-expression analysis. They were placed upstream of SARD1 and CBP60g. Interestingly, both *EDS1* and *PAD4* have been shown to be targets of SARD1 and CBP60g in our ChIP studies. The commonly used defence marker genes *PR1* and *PR2* were also found in one of the clusters co-expressed with SARD1/CBP60g-dependent genes. However, both of them were not identified as binding targets of SARD1 in our ChIP-seq data, suggesting that they are not directly regulated by SARD1 and CBP60g. It is likely that genes co-expressed with SARD1/CBP60g-dependent genes include genes that are either directly or indirectly regulated by SARD1 and CBP60g.

**Figure 9:**
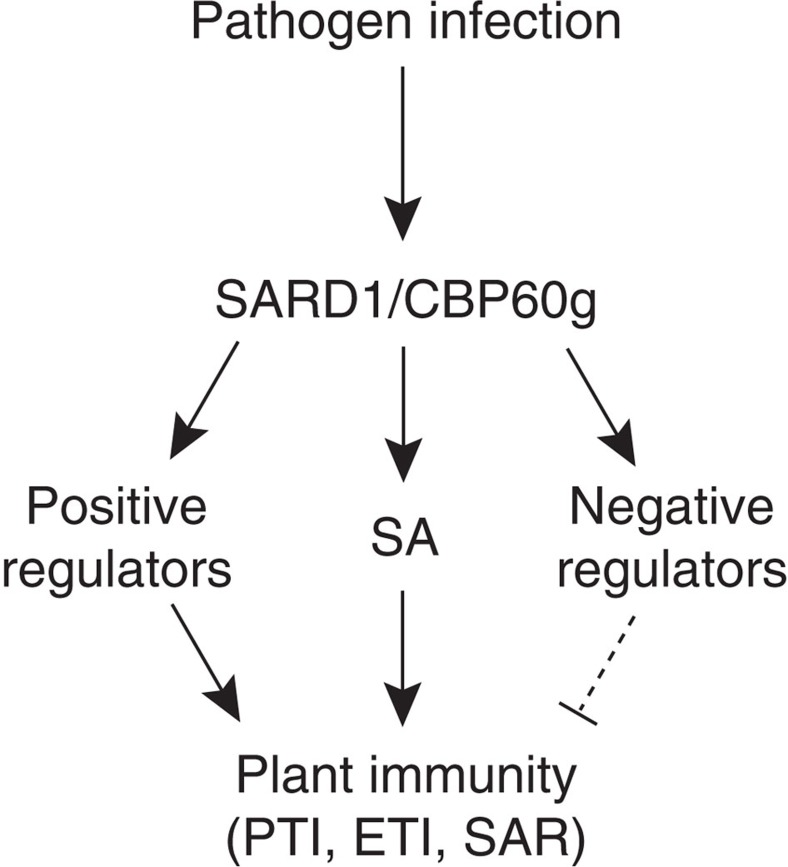
Proposed scheme for regulation of plant defence responses by SARD1 and CBP60g. Following pathogen infection, SARD1 and CBP60g are activated. Subsequently, the expression of a large number of their target genes is turned on. Upregulation of positive regulators of PTI, ETI and SAR and increased SA synthesis lead to enhanced plant immunity against pathogens. Meanwhile, negative regulators of plant immunity are turned on to attenuate plant defence responses.

In summary, a large number of genes encoding key regulators of plant immunity are direct binding targets of SARD1 and CBP60g and their expression is modulated by SARD1 and CBP60g during plant defence. This is consistent with the functions of these two transcription factors in PTI, ETI and SAR. Based on this data we suggest that SARD1 and CBP60g orchestrate the induction of plant defence regulators in plant immunity (Fig. 9).

## Methods

### Plant materials and growth conditions

*Arabidopsis sard1-1*, *cpb60g-1*, *sard1-1 cbp60g-1* and *snc2-1D npr1-1* mutants and *SARD1-HA* and *CBP60g-HA* transgenic plants were described previously^24^,^26^. The *snc2-1D* single mutant was identified from the F2 population of a cross between Col-0 and *snc2-1D npr1-1*. *sard1-1 snc2-1D*, *cpb60g-1 snc2-1D* and *sard1 cpb60g snc2-1D* mutants were isolated from the F2 population of a cross between *sard1-1 cpb60g-1* and *snc2-1D npr1-1*. Primers used for genotyping are listed in Supplementary Table 1. Plants were grown under long day conditions (16 h light per 8 h dark cycle) at 23 °C unless otherwise specified.

### Mutant analysis

To analyze gene expression in *snc2-1D*, *sard1-1 snc2-1D*, *cbp60g-1 snc2-1D* and *sard1 cbp60g snc2-1D*, 50 mg of leaves were collected from three-week-old soil-grown plants for RNA isolation. To analyze gene expression after *P.s.m.* ES4326 infection, leaves of 25-day-old plants grown under short day conditions (12 h light per 12 h dark cycle) were infiltrated with *P.s.m.* ES4326 or 10 mM MgCl_2_ 12 h before sample collection. Four leaves from four individual plants were mixed as one sample. RNA was isolated from three independent samples using EZ-10 Spin Column Plant RNA Mini-Preps Kit (Bio Basic, Canada) and subjected to subsequent RT–PCR analysis. Moloney Murine leukaemia virus reverse transcriptase was used for reverse transcription according to the manufacturer's instructions (New England Biolabs). Real-time PCR was performed using the SYBR Premix Ex Taq II (TAKARA). Primers used for real-time PCR were listed in Supplementary Table 1. *ACTIN1* was used as an internal control.

SA was extracted and measured using a protocol modified from Li *et al*^65^. About 0.1 g of leaf tissue from 3-week-old soil-grown plants was collected and grounded in liquid nitrogen. Four samples for each genotype were collected and analysed. A volume of 0.6 ml of 90% methanol was added to each sample and the samples were subsequently vortexed and sonicated for 20 min. After spinning at 16,000*g* for 20 min, the supernatant was collected and the pellet was re-extracted with 0.5 ml of 100% methanol as above. The supernatants from the two extractions were combined and dried by vacuum. A volume of 0.5 ml of 5% trichloroacetic acid was then added to the dry samples and the samples were vortexed, sonicated for 5 min, and centrifuged at 16,000*g* for 15 min. The supernatant was collected and extracted three times with 0.5 ml of extraction medium (ethylacetate/cyclopentane/isopropanol:100/99/1 by volume). After spinning at 16,000*g* for 1 min, the top organic phases were collected, combined and dried by vacuum. The samples were then re-suspended in 250 μl of mobile phase (0.2 M KAc, 0.5 mM EDTA (pH 5)) by vortexing and sonicating for 5 min. After spinning at 16,000*g* for 5 min, the supernatants were kept and analysed by high-performance liquid chromatography to determine the amount of SA.

*H. arabidopsidis* Noco2 infection assays were carried out on 3-week-old soil-grown plants by spraying plants with *H. arabidopsidis* Noco2 spore suspension at a concentration of 50,000 per ml water. Afterwards, plants were covered with a clean dome and grown at 18 °C under 12 h light per 12 h dark cycle in a growth chamber. *H. arabidopsidis* Noco2 sporulation was scored 7 days later as previously described^66^.

To assay for flg22-induced pathogen resistance, leaves of 4-week-old plants were infiltrated with 1 μM of flg22 or ddH_2_O as control. After 24 h, the same leaves were inoculated with *P. syringae* pv *tomato* DC3000 (OD_600_=0.001) in 10 mM MgCl_2_. Three days post inoculation, a leaf disc was taken from each infected leaf and two leaf discs from the same plant were collected as one sample. The samples were ground, diluted serially in 10 mM MgCl_2_, and plated on Lysogeny broth agar plates with 25 μg ml^−1^ rifampicin and 50 μg ml^−1^ kanamycin. After incubation at 28 °C for 36 h, bacterial colonies were counted from selected dilutions and the colony numbers were used to calculate colony forming units.

### ChIP analysis

For ChIP experiments, two to three fully expanded leaves of 25-day-old plants grown under short day condition were infiltrated with *P.s.m.* ES4326 (OD_600_=0.001). The inoculated leaves were collected after 24 h. About 4 g of leaf tissue was cross-linked in 75 ml of 1% formaldehyde solution plus 0.01% Silwet L-77 under vacuum for 20 min. Glycine (2 M) was added to a final concentration of 0.125 M and the sample was vacuumed for an additional 5 min to stop cross-linking. The tissue was rinsed three times with 60 ml of cold ddH_2_0 and dried with blotting paper. The nuclei were prepared as previously described^67^ and re-suspended in 300 μl of nuclei lysis buffer (50 mM Tris-HCl (pH 8.0), 10 mM EDTA, 1% SDS, 0.1 mM PMSF, 1xPI). The nuclei suspensions were subsequently sonicated to shear the DNA to an average size of 0.3–1 kb.

The sonicated chromatin suspension was spun at 12,000*g* for 5 min at 4 °C to pellet debris. The supernatant was moved to a new 15 ml tube. An aliquot of 5 μl from each sample was moved into a clean 1.5 ml Eppendorf tube and set aside at –20 °C as ‘input'. ChIP dilution buffer (3 ml; 1.1% Triton X-10, 1.2 mM EDTA, 16.7 mM Tris-HCl, pH 8.0, 167 mM NaCl) was then added to the 15 ml tube. For pre-clearing, 100 μl of Protein A agarose beads balanced with the ChIP dilution buffer was added to the chromatin samples and kept at 4 °C for 1 h with rotation. The beads were pelleted at 2,400*g* for 2 min and the supernatants were divided equally into two samples. A volume of 5 μl (0.4 mg ml^–1^) of anti-HA antibody (Roche) was added to one sample for immunoprecipitation and immunoglobin G was added to the other sample as control. The samples were incubated overnight at 4 °C with gentle agitation. Subsequently, 100 μl of Protein A agarose beads balanced with ChIP dilution buffer was added to each sample and kept at 4 °C for 2 h with gentle agitation.

The Protein A beads were then pelleted by centrifugation at 2,400*g* for 10 s at 4 °C. The beads were washed with low salt wash buffer (150 mM NaCl, 0.1% SDS, 1% Triton X-100, 2 mM EDTA, 20 mM Tris-HCl, pH 8.0), high salt wash buffer (500 mM NaCl, 0.1% SDS, 1% Triton X-100, 2 mM EDTA, 20 mM Tris-HCl, pH 8.0), LiCl wash buffer (0.25 M LiCl, 1% NP-40, 1% sodium deoxycholate, 10 mM Tris-HCl, pH 8.0, 1 mM EDTA) and TE buffer sequentially. For each buffer, a quick wash by spinning at 2,400*g* for 10 s and a second wash with 5 min agitation were performed. A volume of 1 ml of buffer was used in each wash.

After the final wash, the samples were pelleted for an additional 2 min at 2,400*g* to remove the supernatant thoroughly. To elute the immune complexes, 250 μl of Elution Buffer (1% SDS, 0.1 M NaHCO_3_) was added to the beads. The samples were vortexed briefly and incubated at 65 °C for 15 min with gentle agitation. After spinning at 3,800*g* for 2 min, the supernatant was carefully transferred to a fresh tube. The pellet was eluted one more time with 250 μl of Elution Buffer and the two eluates were combined (a total of ∼500 μl). At the same time, 500 μl of Elution Buffer was added to the input samples collected before immunoprecipitation. A volume of 1 μl of 10 mg ml^–1^ DNase-free RNase A was added to each sample. After incubation at 37 °C for 1 h, 10 μl of 0.5 M EDTA, 20 μl 1 M Tris-HCl (pH 6.5) and 2 μl of 10 mg ml^–1^ proteinase K were added to each sample. The samples were incubated at 45 °C for 1 h and extracted with the same volume of Tris saturated phenol:chloroform:isoamyl alcohol (25:24:1v/v) twice. DNA was then precipitated by adding 0.7 volume isoproponal, 1/10 volume of 3 M NaOAc and 1 μl 2M glycogen and incubating at room temperature for 30 min. DNA was pelleted by spinning for 20 min at 16,700*g*. The DNA pellets were washed with 80% ethanol, dried at room temperature, re-suspended in 50 μl TE buffer, and stored at −20 °C for further use.

For ChIP-sequencing, DNA sequencing libraries were prepared from chromatin immunoprecipitated DNA using a library preparation kit (E6000s, New England Biolabs) according to the manufacturer's instructions and sequenced using an Illumina Genome Analyzer. Tissue from untreated SARD1-HA transgenic plants was used as the negative control. Sequence reads were mapped to *Arabidopsis* genome sequence using Bowtie 0.12.8 (ref. 68). Sequence coverage at each position on the genome was scored by Samtools^69^ and used to identify peaks in the genome. The 500 bp sequences centred on the peak summits shown in Supplementary Data 1 were used to identify conserved SARD-binding motifs using DREME (ref. 70). DREME was run with default settings and sequences from the promoter regions of randomly chosen genes were used as background control. Confirmation of ChIP-seq results was carried out with three independent ChIP experiments. Independently grown plants were used in each repeat and immunoprecipitated DNA was quantified by real-time PCR using gene-specific primers. The primers used to amplify the promoter regions of the target genes are listed in Supplementary Table 1. Real-time PCR was performed in 96-well format using Bio-Rad CFX connect Real-Time PCR systems and the SYBR Premix Ex Taq II (TAKARA).

### Promoter activity assay

The *NOS101-Luciferase* reporter vector was created by modifying pGreen0229 to include a firefly luciferase gene driven by a basal promoter of the nopaline synthase gene (−101 to +4, designated NOS101). The wild type and mutant versions of the 56 bp promoter fragment of *ICS1* were synthesized and inserted upstream of the NOS101 basal promoter in the reporter vector. Promoter activity assays were performed by expressing the reporter constructs with the 35S-SARD1 construct or empty vector in *Arabidopsis* protoplasts. A 35S-driven Renilla luciferase reporter was included in the assays as internal transfection controls. Transformed protoplasts were incubated for 16–20 h before the activities of the luciferases were measured using a Dual-Luciferase Reporter Assay (Promega).

## Additional information

**Accession codes:** The ChIP-seq data is deposited in the NCBI Sequence Read Archive with accession number: SRX1371906.

**How to cite this article:** Sun, T. *et al.* ChIP-seq reveals broad roles of SARD1 and CBP60g in regulating plant immunity. *Nat. Commun.* 6:10159 doi: 10.1038/ncomms10159 (2015).

## Supplementary Material

Supplementary Figures and Supplementary Table Supplementary Figures 1-2 and Supplementary Table 1

Supplementary Data 1 Candidate target genes containing peaks with heights of 90 or greater in the 1.5 kb region upstream of the translation start site

## Figures and Tables

**Table 1:** Known defence regulators identified as candidate target genes of SARD1 by ChIP-sequencing.

**AGI number**	**Protein name**	**Peak height**
AT3G56400	WRKY DNA-BINDING PROTEIN 70 (WRKY70)	214
AT1G74710	ISOCHORISMATE SYNTHASE 1 (ICS1)	125
AT4G39030	ENHANCED DISEASE SUSCEPTIBILITY 5 (EDS5)	110
AT1G64280	NON-EXPRESSOR OF PATHOGENESIS-RELATED GENES 1 (NPR1)	163
AT1G19250	FLAVIN-DEPENDENT MONOOXYGENASE 1 (FMO1)	99
AT2G13810	AGD2-LIKE DEFENCE RESPONSE PROTEIN 1 (ALD1)	138
AT5G13320	avrPphB SUSCEPTIBLE 3 (PBS3)	199
AT3G48090	ENHANCED DISEASE SUSCEPTIBILITY 1 (EDS1)	258
AT3G52430	PHYTOALEXIN DEFICIENT 4 (PAD4)	137
AT1G33560	ACTIVATED DISEASE RESISTANCE 1 (ADR1)	117
AT4G33300	ADR1-LIKE 1 (ADR1-L1)	324
AT5G04720	ADR1-LIKE 2 (ADR1-L2)	230
AT4G33430	BRI1-ASSOCIATED RECEPTOR KINASE 1 (BAK1)	134
AT2G13790	BAK1-LIKE 1 (BKK1)	190
AT4G34460	ARABIDOPSIS G PROTEIN β-SUBUNIT 1 (AGB1)	200
AT2G39660	BOTRYTIS-INDUCED KINASE 1 (BIK1)	99
AT4G08500	MAPK/ERK KINASE KINASE 1 (MEKK1)	135
AT1G51660	MITOGEN-ACTIVATED PROTEIN KINASE KINASE 4 (MKK4)	141
AT3G45640	MITOGEN-ACTIVATED PROTEIN KINASE 3 (MPK3)	264
AT4G09570	CALCIUM-DEPENDENT PROTEIN KINASE 4 (CPK4)	104
AT3G46510	PLANT U-BOX 13 (PUB13)	207
AT1G80840	WRKY DNA-BINDING PROTEIN 40 (WRKY40)	97
AT2G25000	WRKY DNA-BINDING PROTEIN 60 (WRKY60)	138
AT2G04450	NUCLEOSIDE DIPHOSPHATE LINKED TO SOME MOIETY X 6 (NUDT6)	107
AT4G12720	NUCLEOSIDE DIPHOSPHATE LINKED TO SOME MOIETY X 7 (NUDT7)	169
AT1G11310	MILDEW RESISTANCE LOCUS O 2 (MLO2)	100
AT5G61900	BONZAI 1 (BON1)	151
AT3G61190	BON ASSOCIATION PROTEIN 1 (BAP1)	215
AT2G45760	BON ASSOCIATION PROTEIN 2 (BAP2)	314

AG1, Arabidopsis Genome Initiative; ChIP, chromatin immunoprecipitation; SARD1, SAR DEFICIENT1.
