# Supplementary material for: ChIP-seq reveals broad roles of SARD1 and CBP60g in regulating plant immunity
Source: Nat Commun. 2015 Dec 18;6:10159. doi: 10.1038/ncomms10159 (PMC4703862; doi:10.1038/ncomms10159)
Supplement: Supplementary Figures and Supplementary Table — Supplementary Figures 1-2 and Supplementary Table 1 [file ncomms10159-s1.pdf]

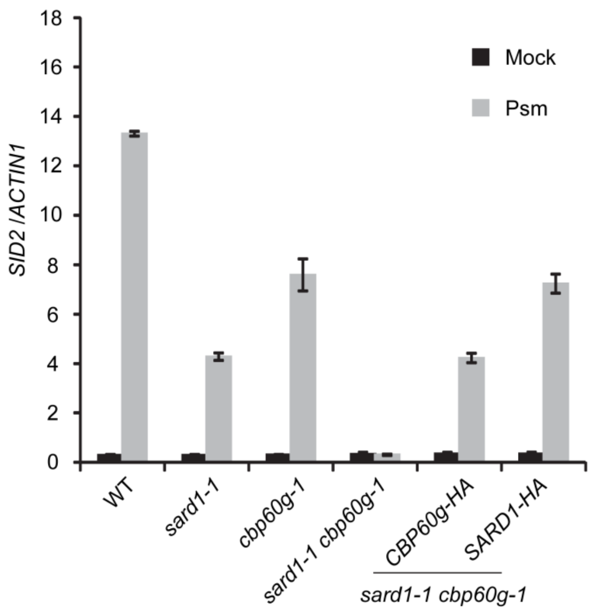

Supplementary Figure 1. Expression levels of *SID2* in wild type, *sard1-1*, *cbp60g-1*, *sard1-1 cbp60g-1* and representative *SARD1-HA* and *CBP60g-HA* transgenic lines in *sard1-1 cbp60g-1* background.

Samples were collected from plants of indicated genotypes 24 hours after inoculation with *P.s.m.* ES4326 (OD_{600} = 0.001) or 10mM $MgCl_2$ (mock). *SARD1-HA* and *CBP60g-HA* transgenic lines were generated by transforming *sard1-1 cbp60g-1* with constructs expressing SARD1-HA or CBP60g-HA under their own promoters. Expression levels of *SID2* were determined by quantitative RT-PCR and normalized with *ACTIN1*. Bars represent means ± s.d. (n = 3).

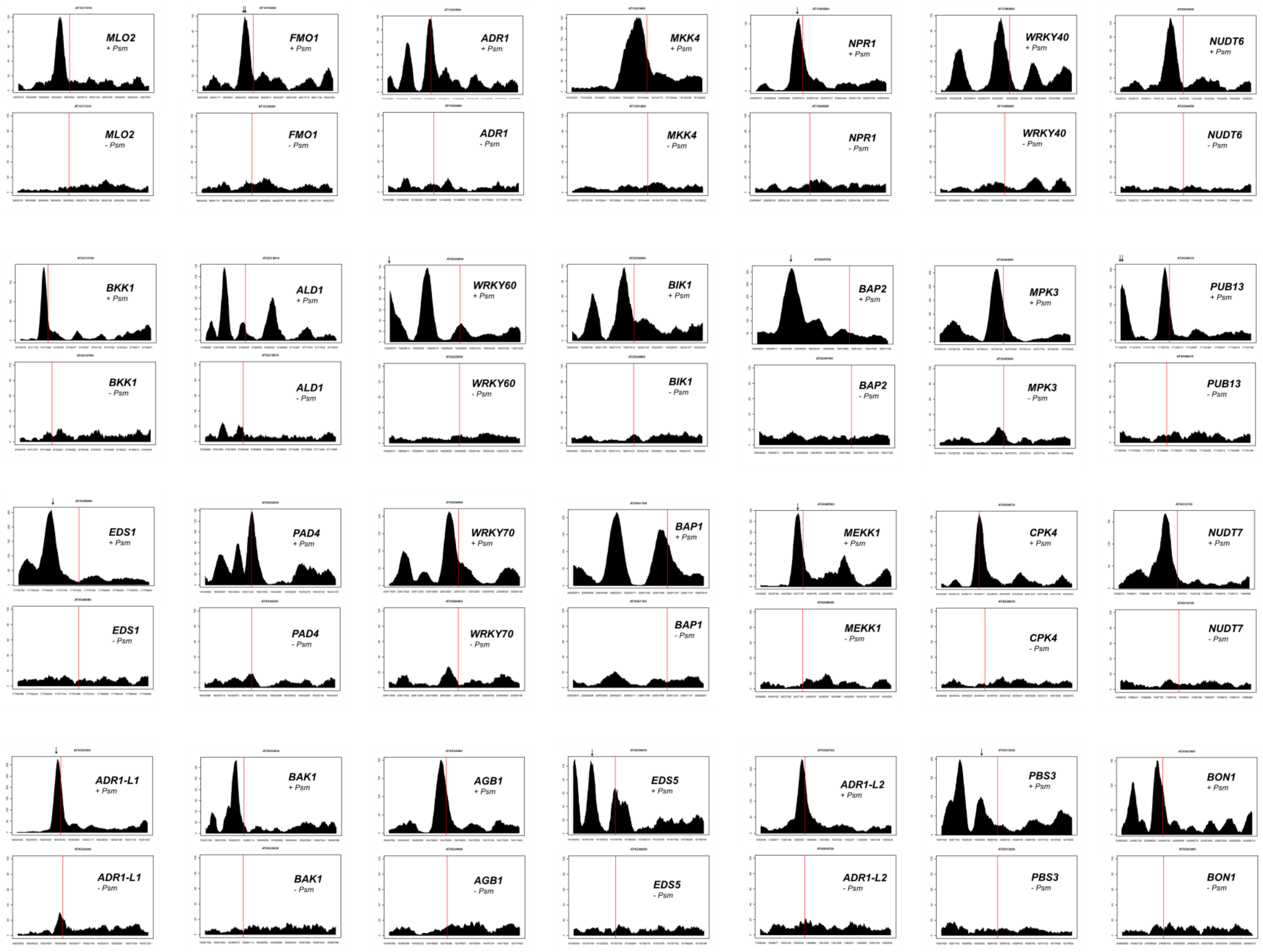

Supplementary Figure 2. Distribution of sequence reads in the promoter and coding regions of genes in Supplementary Data 1.

The vertical axis shows the number of sequence reads. The horizontal axis shows the positions on the chromosomes. Positions of the translation start sites are marked with read lines. Positions of GAAATTT motifs that are close to sequence peaks are indicated by arrows. +*Psm*, distribution of sequence reads from SARD1-HA transgenic plants infiltrated with *P.s.m.* ES4326. – *Psm*, distribution of sequence reads from untreated SARD1-HA transgenic plants.

Supplementary Table 1. Primers used in this study.

Primers for genotyping

mutant	primer name	primer sequence (5'-->3')
sard1-1	SALK_138476-F	CTTCAGTGTCGGAGTAGTCG
	SALK_138476-R	CAAGACCTCTCTAACCTAAC
cbp60g-1	SALK_023199-F	TGTTTCGGTGGACTTGTGAC
	SALK_023199-R	TAAATCCCTCAACGGTCCAG
snc2-1D	snc2-1D-F	TCATCCATTCCAGAGCTTAC
	snc2-1D-mutR	AACACAAGTGAAGAAAGTgT
	snc2-1D-WTR	AACACAAGTGAAGAAAGTgC
npr1-1	npr1-1-WT-F1	GAGGGGATATACGGTGCTaC
	npr1-1-mut-F2	GAGGGGATATACGGTGCTaT
	npr1-1-R1	AGCAGCGTCATCTTCAATTC

Primers for real time RT-PCR, which were designed to amplify 130- to 300-bp DNA fragments.

Gene ID	primer name	primer sequence (5'-->3')
AT2G37620	ACTIN1-F	CGATGAAGCTCAATCCAAACGA
	ACTIN1-R	CAGAGTCGAGCACAATACCG
AT2G14610	PR1 F-2	AGGCAACTGCAGACTCATAC
	PR1 R-2	TTGTTACACCTCACTTTGGC
AT3G57260	PR2-A	GCTTCCTTCTTCAACCACACAGC
	PR2-B	CGTTGATGTACCGGAATCTGAC
AT1G74710	SID2-F101-RT	GTCGTTCGGTTACAGGTTCC
	SID2-R102-RT	ATTAAACTCAACCTGAGGGAC
AT3G56400	WRKY70-RT-FP	GCCAAATTCCCAAGAAGTTAC
	WRKY70-RT-RP	CTTGTGATCTTCGGAATCCAT
AT4G39030	EDS5-F101-RT	GCCAAACAGGACAAGAAAGAAG
	EDS5-R102-RT	GCCGAAACAATCTGTGAAGC
AT1G64280	NPR1-F101-RT	TTACTCTCTATCAGAGGCAC
	NPR1-R102-RT	AAGCACCGTATATCCCCTCG
AT1G19250	FMO1-F101-RT	GGAGATATTCAGTGGCATGC
	FMO1-R102-RT	TTTGGTTAGGCCTATCATGG

AT2G13810	ALD1-F101-RT	TTCCCAAGGCTAGTTTGGAC
	ALD1-R102-RT	GCCTAAGAGTAGCTGAAGACG
AT5G13320	PBS3-F101-RT	CTAAGTTCTGGAACTTCTGG
	PBS3-R102-RT	CATGACTGAAGCAAAGATGG
AT3G48090	EDS1-F1-RT	GATTATTCAGGTGATCGAGC
	EDS1-R2-RT	AATCTGCGGTATCGAGTTGC
AT3G52430	PAD4-F101	CTCTTTCTTCAGTTAAAGATCAAGG
	PAD4-R102	TAGTGTCCGTACCTCTGATG
AT1G33560	ADR1-RT-FP	CTTCATACAGGGGAAATGGA
	ADR1-RT-RP	CAGGAGACATGCCATTGTTG
AT4G33300	ADR1-L1-RTF	GCTATATGCTTGCCCTGAGC
	ADR1-L1-RTR	CAGCAGAGCTCGGTCTATCC
AT5G04720	ADR1-L2-RTF	TTGAAGCTGCGGAAAAATCT
	ADR1-L2-RTR	GAGCAAGAACGAAGTTTGCAC
AT4G33430	BAK1-4F	GGCCACTAAAGTACCATCAG
	BAK1-RT-RP	TCACCTGCAGGGAGTAATAG
AT2G13790	BKK1-RT-FP	ACTCGACTGGGTGAAAGAGG
	BKK1-RT-RP	TTTGCCATTCTTCCCATCTC
AT4G34460	AGB1-TDNA-F	CTGGACATACTGCTGATGTAC
	AGB1-2479-R	ACAGGTCCGTTCTCACCATC
AT2G39660	BIK1-RT-F	ACTTTGGACTAGCTAGAGAC
	BIK1-RT-R	TTCTTCAGGTAGGTACTGTG
AT4G08500	MEKK1-RT-FP	TTTGGATTGGCAAAGGTTTC
	MEKK1-RT-RP	TCAGAGTAGGGGATCTGACCA
AT1G51660	MKK4-RT-FP	AGCCAGAACAGGTCTCCTCA
	MKK4-RT-RP	GAGACCCTCCATGATTAGCAA
AT3G45640	MPK3-RT-FP	AAGAATCACTGTTGAACAAGCTC
	MPK3-RT-RP	CCGTATGTTGGATTGAGTGC
AT4G09570	CPK4-RT-FP	TGGATGCGGCGGATATAGA
	CPK4-RT-RP	TTGTTGAAGCTCGTCAATGG
AT3G46510	PUB13-RTF	CATCAGGGCAGACATACGAA
	PUB13-RTR	TAAACTGCTCGGAGGCTTTG
AT1G80840	WRKY40-RT-FP	TGACACTACCCTCGTTGTGA

	WRKY40-RT-RP	AACACGGACTGATCCTCCAC
AT2G25000	WRKY60-RT-FP	CAAGAAGAAGGTGCAACGAA
	WRKY60-RT-RP	TGGTCAACGAAGAAGCCATT
AT2G04450	NUDT6-105F	TTAGCTGCTAAGTGGATGCC
	NUDT6-105R	TGATCGGTGTTGCAGTAAAG
AT4G12720	NUDT7-RT-FP	CTTGCAAGCTAAGTGGATGC
	NUDT7-RT-RP	GCGATACTTTAAGGCGCTTG
AT1G11310	MLO2-RTF	TTAGACTTGTAGTTGGAGCTGTTG
	MLO2-RTR	GTGTTGGATCCCGAGTGTCT
AT5G61900	BON1-RT-FP	AGAGATGGAGATACTAGACG
	BON1-RT-RP	CGAGACGAGATCATTCATGG
AT3G61190	BAP1-RT-FP	GAACAGGTTCTGGGCATGAT
	BAP1-RT-RP	TAATCTCGGCCTCCACAAAC
AT2G45760	BAP2-RT-F	CCGTTGATACGCACACCAAA
	BAP2-RT-R	GGTGGCTCCGGTGGATTATG

Primers for ChIP-PCR, which were designed to amplify 130- to 300-bp DNA fragments.

**Gene ID**	**primer name**	**primer sequence (5'-->3')**
AT2G37620	ACT1-proF	CCGATTTGATGGAGTCTGGT
	ACT1-proR	CTTCGGCTCACCTTGTTTTC
AT3G56400	WRKY70-P-FP	AAGCAAAAGAAATGGGTGGA
	WRKY70-P-RP	TTTCCTCTTGGTGTGGTTTG
AT4G39030	EDS5-P3-FP	GAGTAGCGTGTGGGCAGAAG
	EDS5-P3-RP	CAATGGCAACAAAAGTCACC
AT1G64280	NPR1-P1-FP	AATGTAAACCGTGGGACGAG
	NPR1-P1-RP	TAAGAATCGGCGAATCCATC
AT1G19250	FMO1-proF2	GCTGGTGTTAGCGGATTAGC
	FMO1-proR2	TGGAAAGTCGGAGAACTCGT
AT2G13810	ALD1-pro #34Fi	TGAATGTTAGTCCTAGTCCC
	ALD1-pro #34Ri	GTGACGCTACAACAACTTTC
AT5G13320	PBS3-ProF2	TTTGCAGAAGTTCCTTGTGG
	PBS3-ProR2	TGTGACGAATTGTTCAGCAAG
AT3G48090	EDS1-pro #37Fi	GATTATCCGGTTTACTTTCC

	EDS1-pro #37Ri	GATTTCGCAGTAACTCAATGA
AT3G52430	PAD4-P1-FP	TGGACCTACCTTTCACAGCA
	PAD4-P1-RP	ACCGAAGCTTGCAACTCACT
AT3G20600	NDR1-proF	TGTTATGGGACATCCCTCGT
	NDR1-proR	CAGTAGGACACACACGCACA
AT1G33560	ADR1-Pro-FP	CTTCGTCTTCTTCGCTATTTTC
	ADR1-Pro-RP	AAGAGTTGCGTCGTGATGTC
AT4G33300	ADR1-L1-ProF	TCGAGCTCCATTGACTTGACT
	ADR1-L1-ProR	ATCGTCGGTCGAATGCTATC
AT5G04720	ADR1-L2-proF	TGGTCCACACTTGCATCATT
	ADR1-L2-proR	AGCTTCGTTGCAGTCACCTT
AT4G33430	BAK1-P-FP	TCTCGGCGTTCCTTCTAATC
	BAK1-P-RP	AAGCTACCACCCACACAAGG
AT2G13790	BKK1-Pro-FP	TCCTTCTTTGGCTCAAATGG
	BKK1-Pro-RP	CGTTGACGAAGACAAACGAA
AT4G34460	AGB1-P-FP	GAGCCGTGTTTGTGTCTTGA
	AGB1-P-RP	AAAAGGGGGAAACTTTGTGG
AT2G39660	BIK1-Pro-FP	CCAAACCCAAAACTCCCTTC
	BIK1-Pro-RP	TCAGCTGCTTGAGCTGAAAA
AT4G08500	MEKK1-Pro-FP	ATCTCTCTCTTCGCCGTCAG
	MEKK1-Pro-RP	ACCAGCTCAAATCCATACGC
AT1G51660	MKK4-Pro-FP	TTAAGCCTGCCACAAACAAA
	MKK4-Pro-RP	TCTCATCGCTTTTGGAATCA
AT3G45640	MPK3-P-FP	AACGAACAGAAACCGTCGAT
	MPK3-P-RP	TGGTTGCTTTGAGGTTTGAA
AT4G09570	CPK4-Pro-FP	TCTTCCTCCTCCTCCTTTGA
	CPK4-Pro-RP	CTAGCTTTTTGCCGAGGAGA
AT3G46510	PUB13-proF	TGGAGTCTCAATCGGGAAAG
	PUB13-proR	GAAATCGCAGCAATCTCGTT
AT1G80840	WRKY40-Pro-FP	CCGATAGAAGCCGAGTTGAG
	WRKY40-Pro-FP	TGGTGGGATTGTTGAAGAGA
AT2G25000	WRKY60-Pro-FP	GACATCCGCCGAAATTGTT
	WRKY60-Pro-RP	AATTTGTTGGCTTGCATCGT

AT2G04450	NUDT6-P-FP	TCCGTTGACAGAGCAAAATG
	NUDT6-P-RP	CCAAGAGGGCGACTTAAAGA
AT4G12720	NUDT7-proF2	TAAACAAAGGGCACGGAAAC
	NUDT7-proR2	CCTGTGTGTACCTTGGAGAGAA
AT1G11310	MLO2-proF	CTCTCGTTGCGGGTCTTATC
	MLO2-proR	CGAAGAGAAAGAGAGACACAGTCA
AT5G61900	BON1-Pro-FP	TGGGTCCCATTTACTGCTCT
	BON1-Pro-RP	GGAGCAACAATTCCCCATAA
AT3G61190	BAP1-P-FP	TATCACGCTGCTGGATTCAA
	BAP1-P-RP	TCTCGCGAATCACGATGTTA
AT2G45760	BAP2-P-FP	GCCGTATCAACGACTCCATT
	BAP2-P-RP	ATACGCATGCATTCACGAAA
